# Karate interventions in children and adolescents: a scoping review of psychosocial and cognitive outcomes

**DOI:** 10.3389/fspor.2026.1840595

**Published:** 2026-08-26

**Authors:** Timea Torzsa, Rebecca Cseh, Judit Futo, Judit Balazs

**Affiliations:** 1 Doctoral School of Psychology, Eotvos Lorand University, Budapest, Hungary; 2 Institute of Psychology, Eotvos Lorand University, Budapest, Hungary; 3 Private Practice, Budapest, Hungary; 4 Oslo New University College, Oslo, Norway; 5 Lumos Child-, Adolescent and Adult Psychiatry Outpatient Clinic, Budapest, Hungary

**Keywords:** children, cognitive outcomes, karate, psychosocial outcomes, scoping review, traditional martial arts, youth

## Abstract

Karate is one of the most widely practiced forms of martial arts among children and adolescents worldwide. Although its potential psychological benefits are recognized, empirical evidence remains limited and heterogeneous. The present scoping review aimed to map the existing literature on karate interventions targeting psychosocial and cognitive outcomes in school-aged children and adolescents. Four electronic databases (ERIC, PubMed, Scopus, and APA PsycINFO) were searched between March and April 2025, and the search was updated in July 2026. Thirteen studies met the inclusion criteria. Included studies consisted of randomized controlled trials, two pre-post studies, and one single-subject study. Risk of bias was assessed using the Cochrane Risk of Bias tools (RoB 2.0, ROBINS-I) to provide contextual information on study quality. Findings were synthesized descriptively and organized according to population type and psychosocial and cognitive outcome domains. The included studies reported a range of outcomes, including resilience, stress, behavioral problems and executive functioning. However, the available evidence is heterogeneous and characterized by methodological limitations. In addition, a substantial proportion of studies focused on clinical or special populations, which should be considered when interpreting the findings. Although several studies reported improvements across psychosocial and cognitive outcomes, conclusions regarding the effectiveness of karate interventions cannot yet be drawn. The review protocol was registered in PROSPERO (CRD42025648442), and reporting followed PRISMA-ScR guidelines.

## 1 Introduction

Martial arts as a form of sport and exercise have become a subject of scientific interest due to their potential role in psychological well-being for all ages (1, 2). The term “martial arts” is broadly applied to a variety of disciplines including traditional martial arts like karate, taekwondo, judo, jiu-jitsu, tai chi and modern styles like mixed martial arts (MMA) (3). Studies suggest that as a result of its qualities and emphasis on self-regulation, martial arts training may be used as a sports-based mental health intervention (4). In addition to investigating the positive effects of martial arts on physical fitness and motor coordination (5), attention has been directed towards identifying its benefits on social, emotional and cognitive development of children and adolescents (6). There has been research focusing on their positive impact on reducing aggression (7), promoting resilience (8), and enhancing cognitive functions such as attention and working memory (9, 10). Martial arts are being used as a form of complementary therapy in several settings for young people (11, 12). Integrating Eastern martial arts into Western physical education systems has been analyzed over the last few decades (13), however, overall evidence on the psychosocial benefits of martial arts and combat sports in youth are still ambiguous (14). In the past years several reviews specifically focused on examining the social, emotional and cognitive benefits of different traditional disciplines (e.g., taekwondo, judo, tai chi) on children and adolescents (15–17), as well as investigating their benefits on specific groups, like children with neurodevelopmental disorders (18). Traditional forms of martial arts such as taekwondo can have a positive impact on the psychosocial factors of trainees (15), while tai chi and qigong's effect on psychological wellbeing and behavior in youth is inconclusive (17). Karate is one of the most widely practiced martial arts worldwide and beyond its physical components, it incorporates elements related to mental and psychological processes (19). It also emphasizes bodily awareness and breathwork, which has similarities to mindfulness (20). It has been proposed that karate is a combat sport, martial art, and self-defence system in one (19). Karate-based programs have increasingly been implemented in educational and clinical settings involving children (21), yet there has been no comprehensive synthesis specifically mapping psychosocial and cognitive outcomes in the literature to date. The existing studies are highly heterogeneous in terms of populations, intervention characteristics and outcomes assessed, highlighting the need to map the available evidence. In this review, psychosocial outcomes include constructs related to psychological well-being, behavioral functioning, and social functioning, whereas cognitive outcomes refer to measures related to executive functioning, attention, and other cognitive processes.

**Table 2:** Characteristics of karate interventions included in the review.

Study	Karate style	Karate components	Additional components	Instructor qualification	Training dose
Parsamajd & Teymori, 2024 (22)	Not reported	Heian Shodan kata	Video demonstration, individualized instruction	Two karate coaches, both holding a second-degree black belt	once per week over a period of 12 weeks (12 sessions in total)
Movahedi et al., 2013 (23)	Not reported	Heian Shodan kata	Video modelling, ASD-adapted teaching strategies, TARGET model	Certified karate instructors trained to teach individuals with ASD	1 session/day, 4 days/week for 14 weeks (56 sessions)
Conant et al., (24) 2008	Kempo	Kata (forms training)	Not reported	Not reported	10-week, 1 h/week
Palermo et al., 2006 (25)	Wa Do Ryu	Kihon, kata, ippon kumite	Breathing tecniques, Training with typically developing peers	Not reported	10 months, three lessons per week
Pinto-Escalona et al., 2024 (26)	Not reported	Not reported	Karate-based PE program	Not reported	2 h/week during one academic year
Bahrami et al., 2015 (27)	Not reported	Heian Shodan kata	Video modelling, ASD-adapted instruction strategies	Certified karate instructors trained to teach individuals with ASD	56 sessions (4 days/week, 14 weeks)
Greco et al., 2019 (28)	Shotokan	Kihon, kumite, kata	Psychoeducational activities	Black-belt Shotokan karate instructor (2nd Dan) + psychologist	12 × 60 min sessions, once per week for 12 weeks
Yildiz & Günel, 2022 (29)	Shotokan	Kihon, kumite, kata	Not reported	2nd Dan karate instructor	75 min, 3 days a week on non-consecutive days for 8 weeks
Greco & Ronzi, 2020 (30)	Shotokan	Heian Shodan kata, kihon, meditation	Dynamic interactions, “partner-drilling” with typically-developing peers	Not reported	Two times per week with each training session lasting 45 min for 12 weeks
Greco, Cataldi, & Fischetti,2019 (31)	Shotokan	Kihon, kumite, kata	Psychoeducational activities	Two black-belt Shotokan karate instructors (at least 2nd Dan)	90 min, once per week for 12 weeks
Gökdere et al., 2025 (33)	Not reported	Pinan Nidan kata	Not reported	Karate instructor (5th Dan)	Three sessions per week, with each session lasting 40 min for 8 weeks
Castillo et al., 2024 (32)	Kenpo	Not reported	Not reported	Not reported	Five times a week for 10 months
Kurniawan et al., 2022 (34)	Not reported	Basic kihon techniques (oi-zuki chudan/jodan, gedan-barai, age-uke, uchi-ude-uke, soto-ude-uke)	Not reported	Not reported	6-week karate training, 18 sessions (30–45 min/session)

The aim of this scoping review was to map the existing empirical studies on karate-based interventions targeting psychosocial and cognitive outcomes in children and adolescents. Specifically, the review aimed to: (1) identify and describe studies examining karate interventions in youth populations, (2) map the psychosocial and cognitive outcomes reported in these studies, and (3) summarize the characteristics of the interventions.

## 2 Method

### 2.1 Protocol

The protocol was originally registered in PROSPERO as a systematic review (CRD42025648442). Due to the heterogeneity of the identified studies regarding participant populations, intervention characteristics, and outcome measures, the review was ultimately conducted and reported as a scoping review following the PRISMA-ScR guidelines. In addition, although the original protocol planned to exclude studies involving neurological disorders (e.g., epilepsy), this criterion was not applied in the final review. Studies involving neurological and neurodevelopmental conditions were retained because these populations represented a substantial proportion of the available evidence. These deviations from the original protocol are acknowledged in the Limitations section.

### 2.2 Inclusion criteria

Eligible studies reported psychosocial or cognitive outcomes (e.g., social, emotional or cognitive functioning). Peer-reviewed articles were prioritized; however, theses and dissertations were also considered during screening. No grey literature sources met the final inclusion criteria following full-text assessment. The age of participants ranged between 5 and 18 years, and only studies published in English were included.

### 2.3 Exclusion criteria

Observational, cross-sectional and case studies were excluded. Studies focusing exclusively on physical outcomes were excluded. Studies involving participants with prior karate or martial arts experience before the intervention were excluded. Given the limited number of identified studies, no restrictions were applied regarding clinical or non-clinical populations, and studies including participants with neurodevelopmental conditions were retained. Although the original protocol planned to exclude studies involving neurological disorders (e.g., epilepsy), studies involving participants with neurodevelopmental conditions (e.g., autism spectrum disorder) were retained because they represented a substantial proportion of the available evidence.

### 2.4 Search strategy

A literature search was conducted between March and April 2025 and updated in July 2026. To identify potentially relevant studies, the following sources were searched: PubMed, Scopus, APA PsycINFO, ERIC, and Google Scholar. The complete search strategy for each database is provided in Supplementary File 1. Search results were exported into Zotero, and duplicate records were removed. Google Scholar results were sorted by relevance, and the first 200 records were manually reviewed to identify potentially eligible studies, as recommended by Haddaway et al. (47). Relevant records were compared with the database search results to identify duplicates. The search strategy combined the following keywords: (karate OR kata OR “karate-do” OR shotokan OR “wado-ryu” OR kyokushin OR kempo OR “martial arts”) AND (child OR adolesc* OR youth OR teen* OR student* OR “school-aged”) AND (psych* OR cognitive OR social OR emotional OR wellbeing OR “well-being” OR behavior* OR behaviour* OR “executive function*” OR “self-esteem” OR “self esteem” OR attention OR resilience). Search terms were adapted to the specific requirements of each database.

**Figure 1:**
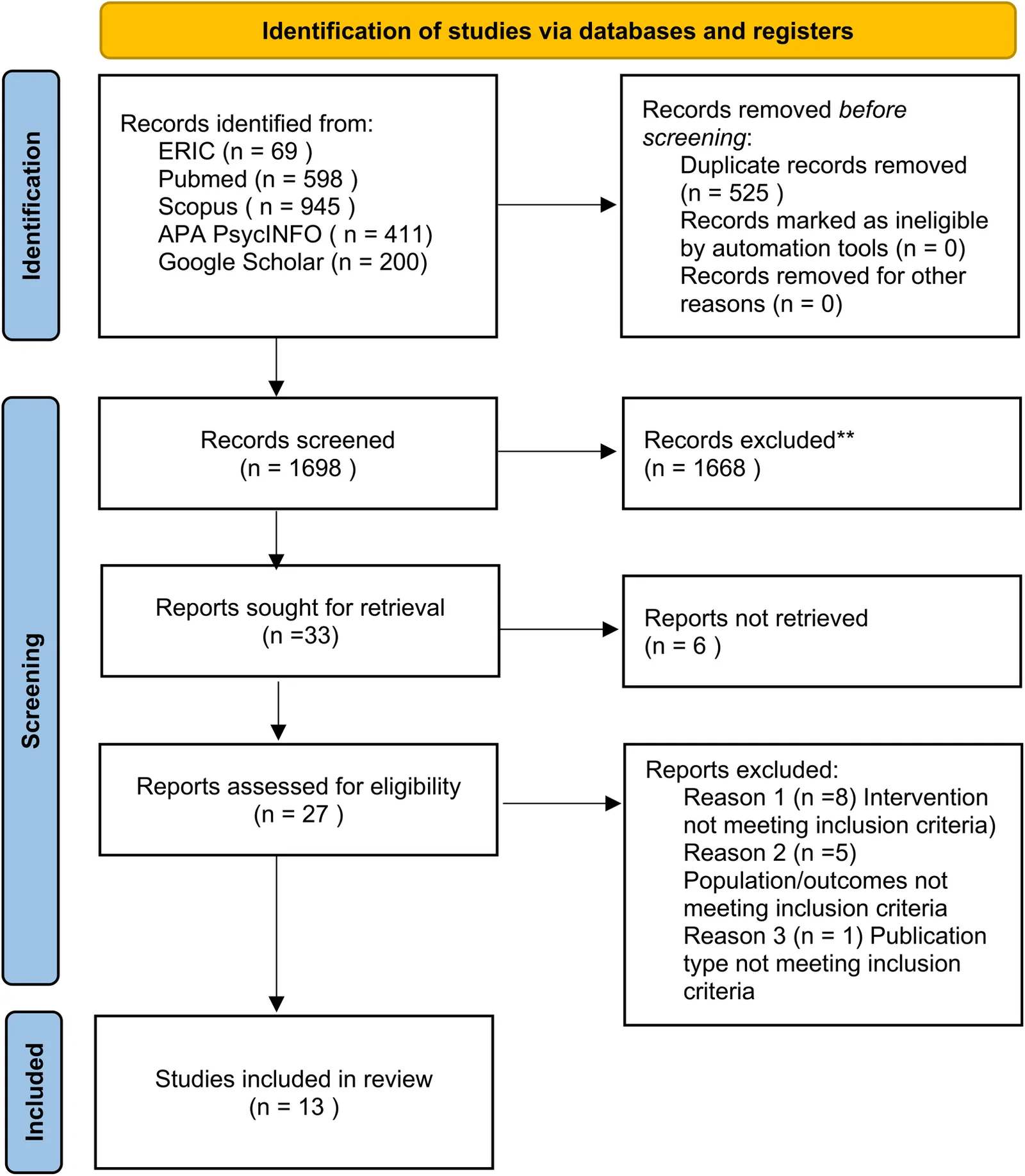
Flow diagram of the review process based on PRISMA recommendations.

The search strategy was developed jointly by the first and second authors. Title and abstract screening was conducted by the first author, who also assessed records for eligibility. No automation tools were used during the screening process. Data extraction for all included full-text studies was conducted independently by both the first and second authors. Any discrepancies were resolved through discussion until consensus was reached. The summary of the search and study selection process is presented in the PRISMA flow diagram (Figure 1). Titles and abstracts were screened against the eligibility criteria, followed by full-text assessment of potentially eligible studies. Extracted data included information on the main characteristics and findings of each study, summarized in Table 1: (1) Population, (2) Intervention, (3) Comparator, (4) Outcomes, (5) Timing, (6) Setting, and (7) Main findings.

**Table 1:** Summary of PICOTS elements for included studies.

Study (Author, year)	Patient population (age, gender, diagnosis)	Intervention	Comparator	Outcome	Timing	Setting	Main Findings
Parsamajd & Teymori, 2024 (22) (Iran)	*n* = 76 elementary school boys (9–12 y/o) with behavioral problems (e.g., aggression, lack of attention, oppositional defiance) identified using the CBCL	12-week Karate Kata training (60 min/week), featuring warm-up, video demonstration (Heian Shodan Kata), individualized instruction, group practice	Control group received no exercise, continued with daily activities	extrinsic behavioral problems measured by Achenbach Child Behavior Checklist (CBCL)	Baseline and post-intervention	RCT in an elementary school setting	Significant improvements in attention/concentration and reductions in aggression, oppositional defiance, and social maladaptation (all *p* < 0.001; large effect sizes); no significant improvements in controls
Movahedi et al., 2013 (23) (Iran)	*n* = 30 children with Autism Spectrum Disorder (5–16 y/o), from autism institutions	14-week Kata techniques training (Heian Shodan Kata) 4sessions/week taught using adapted instructional strategies for children with ASD	Control group-no physical activity, but learning educational skills with same strategies as exercise group	Social dysfunction measured by the Social Interaction subscale of the Gilliam Autism Rating Scale (GARS-2)	Baseline and post-intervention (14 weeks), and 1-month follow-up	RCT with assessments via observation and caregiver reports	Significant reduction in social dysfunction (*p* < 0.001), maintained at 1-month follow-up; no significant change in controls.
Conant et al., 2008 (24) (United States)	*n* = 9 children (8–16 y/o) with epilepsy, enrolled in a pediatric epilepsy program at a U.S. hospital	10-week Kempo Karate form training (60 min/week)	No formal control group; pre/post intervention design	Self-concept measured by Piers–Harris Children's Self Concept Scale and quality of life by Quality of Life in Childhood Epilepsy inventory (QOLCE),	Baseline and post-intervention (10 weeks)	Pilot pre-post study in a clinical/community setting	Significant improvement in the memory domain of quality of life (*p* = 0.01); all other quality-of-life domains, self-concept, and parental stress showed non-significant improvements.
Palermo et al., 2006 (25) (Italy)	*n* = 16 children (8–10 y/o) diagnosed with Oppositional Defiant Disorder	10-month Wa Do Ryu karate program (3 lessons/week), conducted in mixed classes with typically developing peers. Featuring kihon, kata (group and individual practice, breathing techniques)	Control group: no intervention	Temperament traits (intensity, adaptability, mood regulation) measured with the Carey Temperament Scales	Baseline and post-intervention(10 months)	Pilot RCT in martial arts dojo setting	Significant improvements in intensity (*p* < 0.001), mood (*p* = 0.027), and adaptability (*p* = 0.036) compared with controls
Pinto-Escalona et al., 2024 (26) (Spain)	*n* = 388 control *n* = 333 children (7–8 y/o) from 20 schools across 5 European countries	1-year Karate program, replacing usual PE (2 h/week), focused physical activity using karate exercises	Control group: Standard physical education curriculum	Academic achievement (school grades), psychosocial functioning measured using Strengths and Difficulties Questionnaire (SDQ)	Baseline and post-intervention (1 academic year)	Multi-country cluster RCT in elementary school settings	Small but significant benefits compared to the control group for academic achievement (d = 0.16; *p* = 0.003), conduct problems (d = 0.28; *p* = 0.003), No significant benefits for psychosocial difficulties, emotional symptoms, hyperactivity/inattention, peer problems, or prosocial behaviour (all *p* > 0.05).
Bahrami et al., 2015 (27) (Iran)	*n* = 30 children with Autism Spectrum Disorder, from one of three specialized institutions for youth with ASD (5–16 y/o)	14-week karate training (56 sessions, 4 days/week), using Heian Shodan Kata with adapted instructional strategies	Control group: Educational intervention (cognitive and language-based activities)	Communication deficits measured using the Communication subscale of the Gilliam Autism Rating Scale (GARS-2)	Baseline, post-intervention (14 weeks), and 1-month follow-up	RCT conducted in institutions specializing in autism	Communication deficit significantly decreased (*p* < 0.001) and improvements maintained at 1-month follow-up; no significant change was in the control group.
Greco et al., 2019 (28) (Italy)	*n* = 50 students (14–16 y/o), two high schools	12-week Shotokan Karate-based psychosocial intervention (60 min/week) featuring psychoeducation, warm-up, stretching, kata, and kumite. Led by a karate instructor and psychologist	Control group:no intervention, but offered the same karate program after post-assessment.	Resilience measured using CYRM-28 (Child and Youth Resilience Measure)	Baseline and post-intervention (12 weeks)	RCT, school-based setting, blinding of researchers to group allocation.	Significant improvements in overall resilience and resilience subscales (*p* < 0.01; d = 0.59–1.18); no significant changes in controls.
Yildiz & Günel, 2022 (29) (Turkey)	*n* = 124 sedentary high school students (14–17 y/o)	8-week Shotokan Karate training (3 sessions/week, 75 min/session), led by a karate instructor. Components included warm-up, kihon, kata, kumite, and cooldown	Control group: no intervention	Stress and self-confidence levels measured by Stress Level Scale II and Self-Confidence Scale	Baseline and post intervention (8 weeks)	RCT, high school setting. Researchers blinded to group assignment	Stress levels significantly decreased and self-confidence significantly increased (both *p* < 0.001); no significant changes in control group.
Greco & Ronzi, 2020 (30) (Italy)	*n* = 28 children with autism spectrum disorder(8–11 y/o) from local autism treatment center	12-week Shotokan Karate training(2 × 45 min/week with typically-developing peers and ASD-adapted instruction strategies. Focused on social skills and executive functions	Waitlist control group: no intervention	Measured Social-emotional skills (SSIS-RS) and executive functioning (BRIEF)	Baseline and post intervention (12 weeks)	RCT, implemented in a martial arts academy with parental rating-based outcome assessment	Significant improvements in social skills, executive functioning, and problem behaviours (all *p* < 0.01; large effect sizes); no significant changes in controls.
Greco, Cataldi, & Fischetti,2019 (31) (Italy)	*n* = 100 students, 3 high school (14–16 y/o)	12-week Shotokan karate-based program: 90 min/week including technical practice (kihon, kata, kumite) psychoeducational activities	Control group:no intervention, but offered the same karate program after post-assessment.	Resilience (CYRM-28) and Self-Efficacy (SEQ-C)	Baseline and post intervention (12 weeks)	RCT, school-based setting, blinding of researchers to group allocation.	Significant improvements in resilience and self-efficacy (all *p* < 0.05; Cohen's d = 0.35–1.16); no significant improvements in controls
Castillo et al., 2024 (32)	*n* = 57 predominantly Hispanic students (5–11 y/o)	10 months, 5 times/week Kenpo karate	one-group, pretest–posttest design study	Child Trends Socio-Emotional Teacher's Survey	Baseline and post intervention (10 months)	Quasi-experimental, community-based afterschool setting, teacher-rated outcome assessment.	Significant improvements in persistence, self-control, social competence, and conflict resolution without aggression (all *p* < 0.0001; Cohen's d = 1.10–3.02)
Gökdere et al., 2025 (33)	*n* = 43 school-aged children (8–9 y/o)	8-week karate-do kata training, 3 sessions/week, 40 min/session	Control group, no intervention	Bourdon–Vos Test	Baseline and post-intervention (8 weeks)	RCT, school-based setting, paper-and-pencil attention assessment	Significant improvements in sustained attention and processing speed in the intervention group (all *p* ≤ 0.001; Cohen's d = 0.68–1.64), particularly in low-performing children
Kurniawan et al., 2022 (34)	*n* = 4 boys (8–12 y) with mild–moderate ASD	6-week karate training, 18 sessions (30–45 min/session), basic kihon techniques	Single-subject A–B design	Parent-rated questionnaire (ICD-10/DSM-IV adapted)	2-week baseline, 6-week intervention (weekly assessments)		Improvements in parent-rated social, emotional, and executive functioning were observed across the intervention phase.

Risk of bias in the included randomized controlled trials was assessed using the RoB 2 tool (35), which evaluates bias across five domains: the randomization process, deviations from intended interventions, missing outcome data, measurement of the outcome, and selection of the reported results. The tool classifies risk of bias as low, some concerns, or high. Assessments were conducted independently by two reviewers, and discrepancies were resolved through discussion. For cluster-randomized trials, the RoB 2 tool for cluster designs was applied, including additional considerations regarding the timing of participant identification and recruitment (36). Pre-post studies without a control group were assessed using the ROBINS-I tool (37), which evaluates bias across domains including confounding, selection of participants, classification of interventions, deviations from intended interventions, measurement of outcomes, and selection of reported results. Risk of bias in ROBINS-I is classified on a scale from low to critical. The risk of bias assessment was conducted to provide information on methodological quality and to support the interpretation of findings, which are presented in the Discussion. The assessment was not used to determine study eligibility or exclude studies from the review.

### 2.5 Synthesis of results

Descriptive information on the included studies was first summarized. Findings were then synthesized descriptively and organized according to categories of psychosocial and cognitive outcomes. Outcomes not relevant to psychosocial or cognitive domains (e.g., physical or parent-related outcomes) were not included in the synthesis. Studies involving clinical populations and non-clinical populations were synthesized separately.

## 3 Results

The initial literature search identified a total of 2223 records across four databases and Google Scholar: ERIC (*n* = 69), PubMed (*n* = 598), Scopus (*n* = 945), APA PsycINFO (*n* = 411), and Google Scholar (*n* = 200). After removing duplicate records (*n* = 525), 1,698 records were screened based on title and abstract, of which 1,668 were excluded. The reports sought for retrieval included three records identified through Google Scholar after duplicate removal. Thirty-three reports were sought for retrieval, of which six could not be retrieved. Consequently, 27 full-text articles were assessed for eligibility. Following full-text review, 14 articles were excluded because the intervention did not meet the inclusion criteria (*n* = 8), the population or outcomes did not meet the inclusion criteria (*n* = 5), or the publication type did not meet the eligibility criteria (*n* = 1). A total of 13 studies met the inclusion criteria and were included in this scoping review.

### 3.1 Research design and setting

Ten of the thirteen included studies were randomized controlled trials. In eight studies, control groups received no intervention, while two studies employed active control conditions. Two studies reported offering the intervention to control groups after the trial. Three studies used non-randomized designs, including two pre-post studies without a control group and one single-subject study.

### 3.2 Participant characteristics

Across the thirteen studies, the mean age of participants ranged from 7.4 to 15.32 years. The proportion of female participants ranged from 0% to 77.8%. Seven studies involved clinical or special populations, including children and adolescents with autism spectrum disorder (*n* = 4), epilepsy (*n* = 1), and behavioral problems (*n* = 2). Among the latter, one study included children diagnosed with Oppositional Defiant Disorder, whereas the other recruited children with elevated externalizing behavior based on parent and teacher ratings. The remaining six studies included typically developing children and adolescents, including one elementary school-based sample and three high school student samples. Reported socioeconomic status ranged from mixed to above average.

### 3.3 Intervention characteristics

#### 3.3.1 Duration and frequency

Intervention duration ranged from 6 weeks to one academic year, with most studies (*n* = 6) lasting 12–14 weeks. Training frequency varied from one to five sessions per week, and session duration ranged from 30 to 90 min, with most studies using 60-minute sessions.

#### 3.3.2 Settings

Interventions were delivered in various settings, including schools and martial arts gyms (“dojos”). School-based programs were conducted either during physical education classes or after school hours. Not all studies clearly reported the intervention setting.

#### 3.3.3 Instructional approaches and adaptations

Several studies reported adapting training methods to the needs of the target population. These included instructor training, tailored teaching strategies, and, in some cases, the use of video demonstrations. One study combined karate training with a psychosocial intervention co-led by a psychologist, while another incorporated psychoeducational elements as part of the training. These adaptations were reported primarily in studies involving clinical or special populations.

#### 3.3.4 Training characteristics

Most interventions focused on non-contact karate, with a strong emphasis on kata (predefined sequences of movements). Kumite (sparring) was included in a minority of studies, typically in controlled or non-aggressive formats. Some studies incorporated elements aimed at enhancing attention and concentration, such as breathing exercises, meditation, or formalized rituals. Training formats varied, including group-based practice, individual instruction, and, in some cases, integration with typically developing peers.

#### 3.3.5 Karate styles

Four studies explicitly reported using Shotokan karate, while others implemented Kempo or Wado-Ryu karate. Several studies did not specify the karate style, although kata-based training was commonly used. One study integrated karate-based exercises into a broader physical education curriculum. The characteristics of karate interventions are presented in Table 2.

### 3.4 Assessment measures

Most studies employed validated psychometric instruments to assess psychosocial and cognitive outcomes. Measures included behavioral assessments (e.g., CBCL, SDQ), social and communication measures, and scales assessing resilience, self-efficacy, stress and executive functioning. Several studies focusing on clinical populations used condition-specific instruments, such as the Gilliam Autism Rating Scale (GARS) for autism spectrum disorder and the Quality of Life in Childhood Epilepsy inventory (QOLCE). Attention was assessed using the Bourdon–Vos Test, while teacher-reported social-emotional functioning was assessed using the Child Trends Socio-Emotional Teacher's Survey in one study. Overall, outcomes were assessed using a combination of self-report, parent- or teacher-report questionnaires, and performance-based measures.

### 3.5 Psychosocial and cognitive outcomes

#### 3.5.1 Typically developing children and adolescents

Six studies included typically developing children and adolescents. These studies examined a range of psychosocial and cognitive outcomes, including psychological well-being, behavioral functioning, social functioning, attention, and academic achievement.

Several studies examined constructs related to psychological well-being. Greco et al. (28) and Greco, Cataldi, and Fischetti (31) found increases in resilience and self-efficacy among adolescent participants. Yildiz and Günel (29) reported reductions in stress levels alongside increases in self-confidence. Castillo et al. (32) reported improvements in persistence, self-control, social competence, and non-aggressive peer conflict resolution following a karate intervention in typically developing school-aged children enrolled in an after-school program. Pinto-Escalona et al. (26) reported decreases in externalizing behaviour, while Gökdere et al. (33) found improvements in sustained attention. Pinto-Escalona et al. (26) also observed small but significant improvements in academic achievement.

Overall, studies involving typically developing participants reported improvements across several psychosocial and cognitive domains, although findings varied across outcome measures and interventions.

#### 3.5.2 Clinical and special populations

Seven studies involved clinical or special populations, including children and adolescents with autism spectrum disorder, epilepsy, and behavioral problems. These studies mainly examined psychosocial outcomes, including social functioning, communication, behavioral functioning, and psychological well-being, while several also assessed executive functioning.

Conant et al. (24) reported improvements in self-concept and quality of life in children with epilepsy following karate training.

Studies involving children with autism spectrum disorder reported improvements in social and communication functioning. Movahedi et al. (23) and Bahrami et al. (27) found improvements in communication deficits and social dysfunction. Greco and Ronzi (30) reported improvements in social skills and aspects of executive functioning. Kurniawan et al. (34) also reported improvements in social, emotional, and executive functioning following karate training; however, this study used a single-subject design. Parsamajd and Teymori (22) reported reductions in externalizing behaviour following karate-based interventions, while Palermo et al. (25) observed improvements in temperament-related characteristics.

Overall, several studies in clinical and special populations reported improvements across psychosocial and cognitive outcomes, however, these findings should be interpreted with caution because of the heterogeneity of the populations, interventions, and study designs.

### 3.6 Risk of bias assessments

Overall, the included studies were characterized by moderate to high risk of bias. Across randomized controlled trials, some concerns were identified in multiple domains, particularly in relation to the randomization process and selection of reported results, often due to incomplete reporting. Risk of bias due to missing outcome data was generally low. Most studies relied on self-report or informant-report measures and did not employ blinding, which may have introduced bias in outcome assessment. The pre-post study without a control group was assessed as having some concerns to serious risk of bias across most domains. A common issue across studies was lack of control group, limited reporting, including lack of pre-registration and insufficient methodological detail. Detailed risk of bias assessments for each study are presented in Tables 3, 4.

**Table 3:** RoB-2.0 assessments.

Study	Bias arising from the randomization process	Bias due to deviations from intended interventions	Bias due to missing outcome data	Bias in measurement of the outcome	Bias in selection of the reported result	Overall risk of bias
Parsamajd & Teymori, 2024 (22)	some concerns	some concerns	low risk	some concerns	some concerns	some concerns
Movahedi et al, 2013 (23)	some concerns	some concerns	low risk	some concerns	some concerns	some concerns
Palermo et al, 2006 (25)	some concerns	some concerns	low risk	high risk	some concerns	high risk
Pinto-Escalona et al, 2024 (26)	a: low risk ^a^ ­b: low risk	some concerns	low risk	high risk	some concerns	high risk
Bahrami et al, 2015 (27)	some concerns	some concerns	low risk	some concerns	some concerns	some concerns
Greco et al, 2019 (28)	low risk	some concerns	low risk	high risk	some concerns	high risk
Yildiz & Günel, 2022 (29)	low risk	some concerns	low risk	high risk	some concerns	high risk
Greco & Ronzi, 2020 (30)	some concerns	some concerns	low risk	high risk	some concerns	high risk
Greco, Cataldi, & Fischetti,2019 (31)	low risk	some concerns	low risk	low risk	some concerns	some concerns
Gökdere et al., 2025 (33)	some concerns	some concerns	low risk	some concerns	low risk	some concerns

a a = RoB 2 Domain 1a (bias arising from the randomization process); b = RoB 2 Domain 1b (bias arising from the timing of identification or recruitment of participants in cluster-randomized trials).

**Table 4:** ROBINS-I assessments.

Study	Bias due to confounding	Bias in classification of interventions	Bias in selection of participants into the study (or into the analysis)	Bias due to deviations from intended interventions	Bias due to missing data	Bias in measurement of the outcome	Bias in selection of the reported result
Conant et al., 2008 (24)	serious	low	serious	moderate	moderate	serious	moderate
Castillo et al., 2024 (32)	serious	low	low	low	low	serious	moderate

## 4 Discussion

To our knowledge, this is the first scoping review mapping the literature on karate interventions targeting children's psychosocial and cognitive functioning, as well as the settings in which these interventions have been implemented.

Among typically developing children and adolescents, the included studies reported improvements in psychological and cognitive outcomes, including resilience, self-efficacy, stress, self-confidence, attention, and academic performance. For example, Castillo et al. (32) reported improvements in self-control, social competence, and persistence, whereas Gökdere et al. (33) found improvements in sustained attention following karate training. These findings may be related to the integration of physical and mental components within karate practice (38). As a multidimensional activity, karate may support aspects of adolescents' psychological functioning and well-being (39).

Among clinical and special populations, the included studies reported improvements in psychosocial, behavioral, and cognitive outcomes. Greco and Ronzi (30) found improvements in executive functioning, while Movahedi et al. (23) and Bahrami et al. (27) reported changes in communication and social functioning in children with autism spectrum disorder. Conant et al. (24) reported improvements in self-concept and quality of life in children with epilepsy. Karate interventions were also associated with improvements in behavioral functioning and self-regulation in elementary school-aged children (37;38). It has been suggested that traditional martial arts may provide a structured context for managing anger (40), potentially through the combination of physical activity, social interaction and routine (22). Participation in such structured training environments may also support social functioning, as children engage in cooperative practice and shared activities (41).

These findings are in line with previous research highlighting potential cognitive benefits of martial arts practice (10) and broader evidence on the role of physical activity in cognitive development (42, 43).

Additionally, since karate appears to share components with mindfulness practices, such as attention to bodily awareness and breathing (20), it has been hypothesized that karate training may support self-regulation by encouraging reflection on moment-to-moment experiences (44). Nevertheless, none of the included studies directly examined these mechanisms.

The mechanisms underlying these outcomes remain unclear and are likely multifactorial. Self-regulation may represent one potential mechanism discussed in the literature, as karate training involves attention control, discipline and structured practice. Physical activity more broadly has been linked to neural processes involved in self-regulation, which develop throughout childhood and adolescence (45). In this context, it has been suggested that karate training may contribute to the development of self-control and related aspects of psychological functioning (46). However, these proposed mechanisms were not directly investigated in the included studies and should therefore be regarded as hypotheses. Further research is needed to better understand these processes.

These findings should be interpreted with caution, since several studies were assessed as having some concerns or a high risk of bias, particularly regarding outcome measurement and incomplete reporting. One included study used a single-subject design, for which no formal risk of bias assessment was performed; therefore, its findings should also be interpreted with caution. Furthermore, because more than half of the included studies involved clinical or special populations, these findings should not be generalized to all children and adolescents without caution.

### 4.1 Limitations

The included studies differed considerably in terms of participant characteristics, intervention design, duration and outcome measures, which limits the extent to which findings can be generalized. Importantly, more than half of the included studies focused on clinical or special populations (e.g., autism spectrum disorder, epilepsy, behavioral problems), which should be taken into account when interpreting the results. The review also deviated from the original protocol by being conducted as a scoping review and by including studies involving neurodevelopmental conditions. These changes were made to better reflect the available evidence but may limit comparability with the original protocol. An additional limitation is that studies including participants with previous karate experience were excluded. Although this criterion reduced potential confounding related to prior training, it also limited the inclusion of studies examining the long-term or cumulative effects of karate practice. Therefore, the findings mostly reflect the effects of karate interventions in participants without previous karate experience. In addition, most studies relied on self-report or informant-report measures, which may introduce bias. Future studies could benefit from incorporating a broader range of assessment methods, including performance-based measures of cognitive functioning where feasible. Another limitation of the existing literature is the lack of detailed reporting on how karate interventions are implemented for specific target groups. Although several studies mentioned the use of adapted teaching strategies, these were often not described in sufficient detail. More comprehensive descriptions of intervention components and their theoretical background would support a better understanding of potential mechanisms and facilitate the development of more tailored programs.

In conclusion, this scoping review provides an overview of the existing literature on karate-based interventions in children and adolescents. Although several studies reported improvements in psychosocial and cognitive outcomes, the available evidence remains preliminary and highly heterogeneous. The included studies were often limited by small sample sizes, self- or informant-reported outcomes, incomplete reporting, and some concerns or a high risk of bias. Therefore, conclusions regarding the effectiveness of karate interventions cannot yet be drawn. Future research should prioritize well-designed studies with larger samples, clearer reporting of intervention components and more consistent outcome measures, in order to better understand the potential role of karate in supporting psychosocial and cognitive development in youth populations.
