# Supplementary material for: Karate interventions in children and adolescents: a scoping review of psychosocial and cognitive outcomes
Source: Front Sports Act Living. 2026 Aug 26;8:1840595. doi: 10.3389/fspor.2026.1840595 (PMC13556266; doi:10.3389/fspor.2026.1840595)
Supplement: Supplementary file 1 [file Datasheet1.pdf]

Full text- Excluded studies

Reason 1: Intervention not meeting inclusion criteria

1. Burt, L. D., Riley, N., Parkes, R. J., & Eather, N. (2021). The kick-smart program: a randomised feasibility trial evaluating the feasibility and efficacy of a primary-school based martial arts program integrating mathematics, physical fitness and well-being. *J Educ Train Stud*, *9*(3), 47-57.

2. Giardullo, G., Aliberti, S., Sannicandro, I., Fattore, S., & Ceruso, R. (2024). Karate game: using a playful and participatory approach to enhancing children's social and motor perception during the developmental age. *Physical Education Theory and Methodology*, *24*(4), 539-544.

3. Harwood-Gross, A., Lambez, B., Feldman, R., Zagoory-Sharon, O., & Rassovsky, Y. (2021). The effect of martial arts training on cognitive and psychological functions in at-risk youths. *Frontiers in pediatrics*, *9*, 707047.

4. Marusak, H. A., Borg, B., Morales, A., Carrington Smith, J., Blankenship, K., Allen, J. L., ... & Bluth, M. H. (2022). Martial arts-based curriculum reduces stress, emotional, and behavioral problems in elementary schoolchildren during the COVID-19 pandemic: A pilot study. *Mind, Brain, and Education*, *16*(1), 5-12.

5. Twemlow, S. W., Biggs, B. K., Nelson, T. D., Vernberg, E. M., Fonagy, P., & Twemlow, S. W. (2008). Effects of participation in a martial arts–based antibullying program in elementary schools. *Psychology in the Schools*, *45*(10), 947-959.

6. Fu, X., & Shi, P. (2025). The intervention effect of structured martial arts games on behavioral impairments and motor functions in children with autism spectrum disorder. *Frontiers in Psychology*, *16*, 1660040.

7. Cipra, A., & Hall, L. (2019). Core matters: A bullying intervention pilot study. *RMLE Online*, *42*(4), 1-13.

8. Li, K., Dong, G., & Gao, Q. (2023). Martial arts enhances working memory and attention in school-aged children: a functional near-infrared spectroscopy study. *Journal of experimental child psychology*, *235*, 105725.

Reason 2: Population/outcomes not meeting inclusion criteria

1. Jafari, A., Shahbazi, S., & Jafari, H. (2026). Comparative effects of karate and basketball interventions on social communication and physical fitness in preschool children with autism spectrum disorder. *Research in Autism*, *129*, 202746.

2. Pavlova, J., Bodnar, I., Mosler, D., Ortenburger, D., & Wąski, J. (2019). The influence of karate training on preparing preschool girls for school education. *Ido Movement for Culture. Journal of Martial Arts Anthropology*, *19*(2), 12-20.

3. Pavlova, I., Bodnar, I., & Vitos, J. (2018). The role of karate in preparing boys for school education. *Physical Activity Review*, *6*, 54-63.

4. Vlachos, E. (2015). The benefits of using traditional martial arts as an intervention programme for children with behavioural, emotional and social difficulties.

5. Martyshov, A. V., Goryunova, S. V., Glebov, V. V., & Shastun, S. A. (2017). Influences of training on karate on dynamics psycho-emotional stability and training of schoolboys. *RUDN journal of medicine*, *21*(1), 7-13.

Reason 3: Publication type not meeting inclusion criteria

1. Capulis, S., Dombrovskis, V., & Guseva, S. (2017). Karate-do for cognitively passive pre-schoolers from low-income families. In *ICERI2017 Proceedings* (pp. 114-120). IATED.
