# Supplementary material for: Karate interventions in children and adolescents: a scoping review of psychosocial and cognitive outcomes
Source: Front Sports Act Living. 2026 Aug 26;8:1840595. doi: 10.3389/fspor.2026.1840595 (PMC13556266; doi:10.3389/fspor.2026.1840595)
Supplement: Supplementary file 2 [file Datasheet2.pdf]

Search date: 1 July 2026

Limits: English language, publication years 1990–2026.

ERIC:

(karate OR kata OR "karate-do" OR shotokan OR "wado-ryu" OR kyokushin OR kempo OR "martial arts") AND (child* OR adolesc* OR youth OR teen* OR student* OR "school-aged") AND (psych* OR cognitive OR social OR emotional OR wellbeing OR "well-being" OR behavior* OR behaviour* OR "executive function*" OR "self-esteem" OR "self esteem" OR attention OR resilience)

APA PsycINFO:

(karate OR kata OR "karate-do" OR shotokan OR "wado-ryu" OR kyokushin OR kempo OR "martial arts") AND (child* OR adolesc* OR youth OR teen* OR student* OR "school-aged") AND (psych* OR cognitive OR social OR emotional OR wellbeing OR "well-being" OR behavior* OR behaviour* OR "executive function*" OR "self-esteem" OR "self esteem" OR attention OR resilience)

Scopus:

TITLE-ABS-KEY ((karate OR kata OR "karate-do" OR shotokan OR kyokushin OR "wado-ryu" OR kempo OR "martial arts") AND (child* OR adolesc* OR youth OR teen* OR student* OR "school-aged")AND (psych* OR cognitive OR social OR emotional OR wellbeing OR "well-being" OR behavior* OR behaviour* OR "executive function*" OR "self-esteem" OR "self esteem" OR attention OR resilience))

Pubmed:

(karate OR kata OR "karate-do" OR shotokan OR "wado-ryu" OR kyokushin OR kempo OR "martial arts") AND (child* OR adolesc* OR youth OR teen* OR student* OR "school-aged") AND (psych* OR cognitive OR social OR emotional OR wellbeing OR "well-being" OR behavior* OR behaviour* OR "executive function*" OR "self-esteem" OR "self esteem" OR attention OR resilience)

Google scholar:

(karate OR kata OR "karate-do" OR shotokan OR kyokushin OR "wado-ryu" OR kempo OR "martial arts") AND (child OR adolescent OR youth OR teen OR student OR "school-aged") AND (psychological OR cognitive OR social OR emotional OR wellbeing OR "well-being" OR behavior OR behaviour OR "executive function" OR "self-esteem" OR attention OR resilience)

Due to Google Scholar search limitations, combinations of the above search terms were used. Results were sorted by relevance, and the first 200 records were screened.
